# In vitro and in silico evaluation of antiretrovirals against SARS-CoV-2: A drug repurposing approach

**DOI:** 10.3934/microbiol.2023002

**Published:** 2023-01-16

**Authors:** Maria I. Zapata-Cardona, Lizdany Florez-Alvarez, Ariadna L. Guerra-Sandoval, Mateo Chvatal-Medina, Carlos M. Guerra-Almonacid, Jaime Hincapie-Garcia, Juan C. Hernandez, Maria T. Rugeles, Wildeman Zapata-Builes

**Affiliations:** 1 Grupo Inmunovirologia, Facultad de Medicina, Universidad de Antioquia UdeA, Medellin, Colombia; 2 Institute of Biomedical Sciences, University of Sao Paulo, Sao Paulo, Brazil; 3 Grupo de investigacion GIRYSOUT, Universidad del Tolima, Ibague, Colombia; 4 Grupo de investigacion, Promocion y prevencion farmaceutica, Facultad de ciencias farmaceuticas yalimentarias, Universidad de Antioquia UdeA, Medellin, Colombia; 5 Grupo Infettare, Facultad de Medicina, Universidad Cooperativa de Colombia, Medellin, Colombia

**Keywords:** antiretrovirals, SARS-CoV-2, COVID-19, molecular docking, drug repurposing

## Abstract

**Background:**

Drug repurposing is a valuable strategy for rapidly developing drugs for treating COVID-19. This study aimed to evaluate the antiviral effect of six antiretrovirals against SARS-CoV-2 in vitro and in silico.

**Methods:**

The cytotoxicity of lamivudine, emtricitabine, tenofovir, abacavir, efavirenz and raltegravir on Vero E6 was evaluated by MTT assay. The antiviral activity of each of these compounds was evaluated via a pre-post treatment strategy. The reduction in the viral titer was assessed by plaque assay. In addition, the affinities of the antiretroviral interaction with viral targets RdRp (RNA-dependent RNA polymerase), ExoN-NSP10 (exoribonuclease and its cofactor, the non-structural protein 10) complex and 3CLpro (3-chymotrypsin-like cysteine protease) were evaluated by molecular docking.

**Results:**

Lamivudine exhibited antiviral activity against SARS-CoV-2 at 200 µM (58.3%) and 100 µM (66.7%), while emtricitabine showed anti-SARS-CoV-2 activity at 100 µM (59.6%), 50 µM (43.4%) and 25 µM (33.3%). Raltegravir inhibited SARS-CoV-2 at 25, 12.5 and 6.3 µM (43.3%, 39.9% and 38.2%, respectively). The interaction between the antiretrovirals and SARS-CoV-2 RdRp, ExoN-NSP10 and 3CLpro yielded favorable binding energies (from −4.9 kcal/mol to −7.7 kcal/mol) using bioinformatics methods.

**Conclusion:**

Lamivudine, emtricitabine and raltegravir showed in vitro antiviral effects against the D614G strain of SARS-CoV-2. Raltegravir was the compound with the greatest in vitro antiviral potential at low concentrations, and it showed the highest binding affinities with crucial SARS-CoV-2 proteins during the viral replication cycle. However, further studies on the therapeutic utility of raltegravir in patients with COVID-19 are required.

## 1. Introduction

The current coronavirus disease 2019 (COVID-19) pandemic, caused by severe acute respiratory syndrome coronavirus 2 (SARS-CoV-2), has evolved into a primary public health threat, affecting the life and health of more than 500 million people [1]. Despite the combined efforts to develop vaccines, which are being administered around the world, the appearance of variants with several degrees of resistance to the vaccine and naturally acquired antibodies [2], along with the restricted specific therapy available for this disease, underline the need for continuing with the evaluation of molecules with antiviral properties [3],[4]. This requires a better understanding of the structure of viral particles and the identification of SARS-CoV-2 proteins used as targets for drug design and repurposing [5].

SARS-CoV-2 is an enveloped virus with surface-projected club-like spikes. Each viral particle possesses an unsegmented, single-stranded, positive-sense RNA (+ssRNA) genome with 5′-cap and 3′-poly(A) tail [6]. Two-thirds of the viral genome allows the production of two polyproteins that are proteolytically processed to produce 16 non-structural proteins (NSPs), while the remaining region encodes for four structural proteins (spike, envelope, membrane and nucleocapsid) and nine accessory proteins [7]. Some of these proteins have a valuable role in the viral replicative cycle. For instance, NSP12, also known as RNA-dependent RNA polymerase (RdRp), synthesizes viral RNA [8]. The NSP14 is a bifunctional protein with a putative N-terminal exoribonuclease (ExoN) domain playing a proofreading role in RNA replication and an assumed C-terminal guanine-N7-methyltransferase (N7-Mtase) domain responsible for mRNA capping [9]. NSP10 is a stimulatory factor for increasing the exoribonuclease activity of NSP14 [9],[10]. The 3-chymotrypsin-like cysteine protease (3CLpro) is the leading viral protease responsible for processing polyproteins translated from viral RNA, and it recognizes specific cleavage sites [11],[12].

To date, several studies carrying out virtual screening with databases of antivirals approved by the USA Food and Drug Administration have been published as the first step in identifying promising molecules with therapeutic effects against COVID-19 [13].

Among the spectrum of potential antiviral candidates against SARS-CoV-2, antiretroviral drugs for human immunodeficiency virus (HIV) infection are commonly referenced [14]–[20]. These antiretrovirals are classified according to the phase in which they interfere throughout the viral replicative cycle, and they are mostly grouped as fusion inhibitors, CCR5 antagonists, post-attachment inhibitors, nucleoside reverse transcriptase inhibitors (NRTIs), non-NRTIs (NNRTIs), integrase inhibitors (INI) and protease inhibitors [21]. This drug group is subject to extensive research to determine its effect on COVID-19 [14],[15]. Significant strides have been made, particularly with NRTIs, NNRTIs and INIs against SARS-CoV-2 by using in vitro methodologies and in silico approaches [16]–[18],[22],[23].

Regarding NRTIs, lamivudine (3TC) has shown affinity by SARS-CoV-2 RdRp through in silico experiments [24]. Further, it has been reported that the triphosphates of tenofovir and emtricitabine (FTC) act as terminators for the SARS-CoV-2 RdRp-catalyzed reaction [19]. In addition, tenofovir dixoproxil fumarate (TDF) significantly reduced SARS-CoV-2 particle production in Vero CCL81 cells [25]. Abacavir (ABC) showed high binding affinity by RdRp and 3CLpro to SARS-CoV-2 through molecular docking [26]. This compound was also capable of terminating RNA synthesis catalyzed by the SARS-CoV-2 RdRp in polymerase extension experiments [27].

Additionally, the NNRTI efavirenz showed inhibitory potential against the SARS-CoV-2 3CLpro in the simulation of a molecule transformer-drug target interaction model [16]. This compound also inhibited in vitro the SARS-CoV-2 infection in A549 cells expressing the ACE2 receptor [28]. Finally, it has been proposed that raltegravir (RAL), an INI, could interfere with the activity of RdRp, 3CLpro and NSP14-NSP10, with promising molecular docking results [10],[18].

According to this evidence, our study evaluated the in vitro antiviral activity of six antiretrovirals (3TC, FTC, tenofovir, ABC, efavirenz and RAL) against the D614G ancestral strain of SARS-CoV-2. We also evaluated the binding affinity of each of these drugs with three viral targets (RdRp, ExoN-NSP10 and 3CLpro) to enrich the available evidence of the possible therapeutic effects of these drugs on the COVID-19 treatment.

## 2. Methods

### 2.1. Preparation of antiretroviral compounds

The active compounds of the following five antiretrovirals were donated by a pharmaceutical company: 3TC, FTC, TDF, ABC and efavirenz. RAL was provided by Tech Innovation Group. Compounds were solubilized in dimethyl sulphoxide (Sigma) at a final concentration of 87.2 mM, 121.3 mM, 10.0 mM, 104.8 mM, 44.4 mM or 4.1 mM. For in vitro evaluations, antiretrovirals were used at concentrations ranging from 3.1 to 200 µM, and their biological activities have been reported [29],[30]. Chloroquine (CQ) was bought from Sigma-Aldrich (St. Louis, Missouri, USA) and prepared in phosphate-buffered saline (PBS, Lonza, Rockland, ME, USA) at 15 mM. The optimal CQ concentration (100 µM) was selected according to previous studies [31]. The stock solutions were frozen at −20 °C, except Tenofovir and CQ, which were stored at 4 °C and −80 °C, respectively.

### 2.2. Cells and virus

Cytotoxicity and antiviral assays were done on the Vero E6 cell line (provided by Instituto Nacional de Salud, Colombia). The cultures were incubated at 37 °C and 5% CO_2_. Vero E6 cells were propagated in Dulbecco's Modified Eagle Medium (DMEM, Sigma-Aldrich) supplemented with 2% heat-inactivated fetal bovine serum (FBS), 2 mM L-glutamine (Gibco), 1% Penicillin-Streptomycin (Gibco) and 3.7 g/L sodium bicarbonate (Sigma-Aldrich).

The infections were done with a viral stock of the Colombian isolate, a D614G ancestral strain of SARS-CoV-2 (lineage B.1, EPI_ISL_536399) [32]. All virus manipulation procedures were performed in a biosafety level-3 (BSL-3) laboratory, according to the conditions set out in Biosafety in Microbiological and Biomedical Laboratories [33].

### 2.3. Effects of the compounds on the cellular viability

The 3-[4,5-dimethylthiazol-2-yl]-2,5-diphenyltetrazolium bromide (MTT) assay was used to evaluate the toxicity of six antiretrovirals on Vero E6 cells for 48 h, as previously described [34]. The concentrations evaluated ranged from 12.5 to 200 µM. The absorbance was measured on a Multiskan GO spectrophotometer (Thermo) at 570 nm. Cell viability was calculated based on the viability of the untreated cells (viability control). Concentrations that maintained more than 90.0% of cell viability after treatment were considered non-toxic and used for the antiviral evaluation. CQ (positive control of viral inhibition) was evaluated at 100 µM [35]. Two independent experiments were performed with four replicates (n = 8).

### 2.4. Evaluation of the antiviral effects of the compounds

The antiviral activity of each of the six antiretrovirals against SARS-CoV-2 was evaluated on Vero E6 through the previously described strategy of pre-post treatment [34]. Briefly, Vero E6 cells (1.0 × 10^4^ cells/well) were seeded in 96-well plates. The 3TC was evaluated at 50–200 µM, FTC at 25–100 µM, tenofovir and ABC at 12.5–50 µM, efavirenz at 3.1–12.5 µM and RAL at 6.3–25 µM at 1 h before infection and 48 h post-infection. CQ was a positive control of viral inhibition (100 µM). Infections were done at a 0.01 multiplicity of infection for 1 h. Infectious viral particles in supernatants were quantified by plaque assay [34]. The supernatant of infected cells without treatment was used as the untreated control. Two independent experiments with two replicates per experiment were performed (n = 4).

### 2.5. Plaque assay

Tenfold serial dilutions of the supernatants obtained from antiviral assay were prepared in DMEM with 2% FBS and used to inoculate confluent monolayers of Vero E6 cells in 24-well plates. After 1 h of incubation, the viral inoculum was removed and cells were overlaid with 1.5% carboxymethyl-cellulose (Sigma-Aldrich) in DMEM with 2% FBS. After 3 days of incubation at 37 °C, the monolayers were washed with PBS, fixed with 4% formaldehyde and stained with 2% crystal violet solution (Sigma-Aldrich). Plaques were counted and used to calculate the number of plaque-forming units per milliliter (PFU/mL) [36]. The difference in the viral titer after treatment compared to the untreated control was expressed as inhibition percentage.

### 2.6. Docking molecular

The molecular docking simulation was used to determine the binding energy of six antiretrovirals to the RdRp, ExoN-NSP10 and 3CLpro proteins of SARS-CoV-2. These proteins are necessary for viral RNA replication and polyprotein processing [12]. The crystal structures of RdRp (Identification code, ID: 6M71) [8], ExoN-NSP10 (ID:7MC6) [37] and 3CLpro (ID: 6M2N) [38] were obtained from the Protein Data Bank (PDB) [39]. The resolution structures selected were lower than 3 Å [40]. The proteins were subjected to preparation by using Discovery Studio [41] and AutoDockTools (ADT). The active forms of the antiretrovirals [42] were drawn and optimized by using Avogadro software [43] and ADT. Remdesivir [44],[45], pibrentasvir [46] and CQ [47],[48] were used as positive controls of the interaction with RdRp, ExoN-NSP10 and 3CLpro, respectively.

PrankWeb [49] was used to determine the number of pockets and the amino acid residues that comprise them. This program also described the size (volume), depth, surface area or general hydrophobicity of each pocket (Table 1). In addition, Protein plus [50] was implemented to verify the number of pockets obtained by PrankWeb [49], and to describe their characteristics (size, shapes, amino acids composition and descriptor functional groups). The pockets were selected according to the active site or catalytic domain for each protein, as based on previous reports [16],[51],[52].

Couplings were carried out using AutoDock Vina version 4.2.6 [53], with an exhaustiveness value of 20 and a grid box of 24 Å × 24 Å × 24 Å, centered at (116.7829 Å, 109.9570 Å, 123.9430 Å) (XYZ coordinates) for RdRp (PDB ID: 6M71), (28.6904 Å, −1.9647 Å, 13.6836 Å) for ExoN-NSP10 (PDB: 7MC6) and (−47.585 Å, 1.135 Å, −5.600 Å) for 3CLpro (PDB ID:6M2N) (Table 1). The best docking conformation of protein-ligand interactions was predicted based on the binding energy value (kcal/mol). The docked structures were analyzed and visualized by using BIOVIA Discovery Studio Visualizer 16.1.

**Table 1.** Pocket properties of RdRp, EXON-NSP10 and 3CLpro proteins of SARS-CoV-2.

Target protein	Amino acids making up the pocket	Depth (Å)	Surface (Å2)	Volume (Å3)	Hydrophobicity ratio	Amino acid composition: (apolar, polar, positive, negative amino acid) ratio
RdRp (PDB ID: 6M71)	TRP 617, ASP 618, TYR 619, LEU 758, SER 759, ASP 760, ASP 761, ALA 762, LYS 798, TRP 800, GLU 811, CYS 813, SER 814	14.62	1223.18	839.19	0.54	0.45, 0.33, 0.12, 0.09
ExoN-NSP10 (PDB ID: 7MC6)	ASN 104, PRO 141, GLN 145, PHE 146, TRP 186, ALA 187, PHE 190, GLN 191, ASN 252, LEU 253, GLN 254, ALA 267 HIS 268, ASP 273, MET 276 ASP 90, VAL 91, GLU 92, GLY 93, HIS 95	15.11	361.69	236.12	0.34	0.50,0.28,0.06,0.11
3CLpro (PDB ID: 6M2N)	PHE 140, LEU 141, ASN 142, GLY143, SER144, CYS 145, HIS163, HIS 164, MET 165, GLU 166, ASP 187, GLN 189, THR 25, THR 26, LEU 27, HIS 41, CYS 44, THR 45, MET 49	17.18	762.59	633.1	0.41	0.37,0.37, 0.17,0.10

### 2.7. Statistical analysis

All data were analyzed by using GraphPad Prism (La Jolla, CA, USA). The normality was determined by using the Shapiro-Wilk test. Data are presented as mean ± standard deviation (SD) or median ± interquartile range (IQR), according to normality. Statistical differences were evaluated via the student's t-test or Mann-Whitney U test. A value of p ≤ 0.05 was considered significant, with *p ≤ 0.05, **p ≤ 0.01, ***p ≤ 0.001 and ****p < 0.0001.

## 3. Results

### 3.1. Vero E6 cell viability in the presence of antiretrovirals

**Figure 1.**
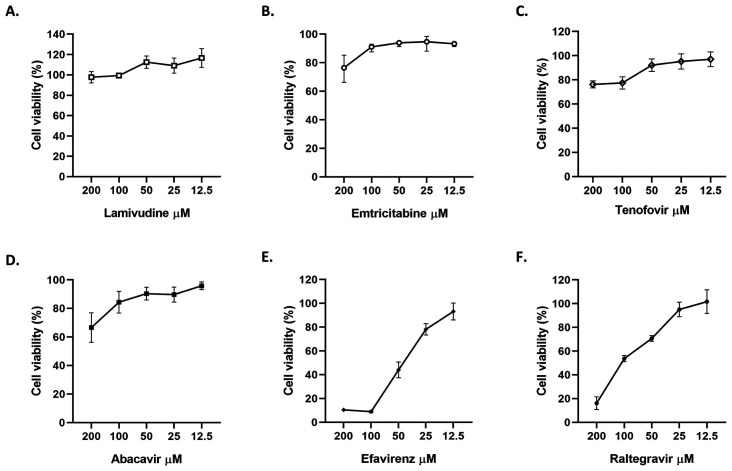
Viability of Vero E6 cells after 48 h of antiretroviral treatment (from 12.5 to 200 µM). Data are presented as mean ± SEM (standard error of mean), according to normality. The viability percentages of the treated cell were calculated based on an untreated control. Two independent experiments with four replicates each were performed (n = 8).

The cytotoxic effects of six antiretrovirals were evaluated by MTT assay. Vero E6 viability was higher than 90% at concentrations under 200 µM of 3TC, 100 µM of FTC, 50 µM of tenofovir and ABC, 12.5 µM of efavirenz and 25 µM of RAL (Figure 1). CQ (control of viral inhibition) was non-toxic at 100 µM.

### 3.2. 3TC, FTC and RAL inhibited infectious SARS-CoV-2 particles in Vero E6 cells

**Figure 2.**
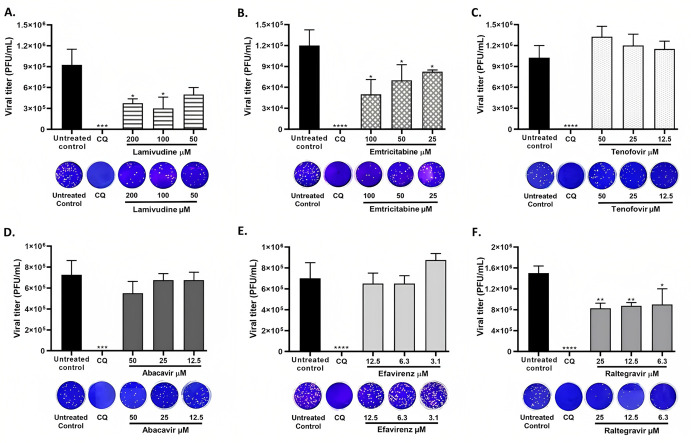
3TC, FTC and RAL inhibited infectious SARS-CoV-2 particles on Vero E6 cells. SARS-CoV-2 titer (PFU/mL) obtained after pre-post treatment with A. 3TC, B. FTC, C. Tenofovir, D. ABC, E. Efavirenz or F. RAL on Vero E6 cells. CQ was used as a positive control of viral inhibition (100 µM). Data are presented as median ± IQR (n = 4). Mann-Whitney test: *p ≤ 0.05, **p ≤ 0.01, ***p ≤ 0.001, ****p < 0.0001. In addition, representative images from the plaque assay of each treatment are shown.

A pre-post treatment strategy was performed for the antiviral evaluation of six antiretrovirals against SARS-CoV-2. Two NRTIs (3TC and FTC) demonstrated an anti-SARS-CoV-2 effect, as shown in Figure 2A. Inhibition percentages of 58.3% (p = 0.03) and 66.7% (p = 0.03) were obtained after the treatment with 3TC at 200 µM and 100 µM, respectively. FTC also inhibited the viral infection at 100 µM (59.6%, p = 0.03), 50 µM (43.4%, p = 0.03) and 25 µM (33.3%, p = 0.03) (Figure 2B). Further, RAL showed anti-SARS-CoV-2 activity at 25 µM (43.3%, p = 0.004), 12.5 µM (39.9%, p = 0.004) and 6.3 µM (38.2%, p = 0.01) (Figure 2F).

In contrast, the viral titer of SARS-CoV-2 was not significantly reduced by tenofovir, ABC or efavirenz treatment at any of the tested concentrations (Figure 2C–E). The positive control of viral inhibition (CQ) inhibited SARS-CoV-2 at 100 µM (100%, p < 0.0001).

### 3.3. Identification and characterization of the viral protein binding site

RdRp, also known as NSP12 (PDB ID: 6M71), showed 37 pockets, with values between 1.00 and 8.79. We selected the pocket 20 (value of 2.54) because, although it did not obtain the highest score, it included amino acids from the active site of the enzyme (SER 759, ASP 760 and ASP761) [51]. On the other hand, the ExoN-NSP10 complex (PDB ID: 7MC6) showed six pockets (values from 1.23 to 16.41). We selected the pocket with the highest score (16.41) to evaluate the interaction because it comprised amino acid residues located in the exonuclease domain of NSP14 (ASP 90, GLU 92, GLU 191, HIS 268 and Asp273) [52]. The complex with NSP10 was necessary due to the ExoN domain of NSP14 needing this protein to catalyze nucleotide excision efficiently [54]. Finally, 3CLpro (PDB ID: 6M2N) showed 15 pockets (values between 1.06 and 12.65). The pocket with the highest score (12.65) was selected to evaluate the interaction because of the included amino acids related to the active catalytic domain (CYS 145 and HIS 41) of 3CLpro [16]. The chemical properties and size descriptions of these pocket sites are shown in Table 1.

### 3.4. Antiretrovirals showed favorable binding energies with three SARS-CoV-2 proteins, as calculated by molecular docking

Favorable binding energies and coupling were obtained between six antiretrovirals and three non-structural proteins of SARS-CoV-2 by using molecular docking (Table 2). Each complex was analyzed to identify the amino acids of the viral proteins that interacted with the compounds.

**Figure 3.**
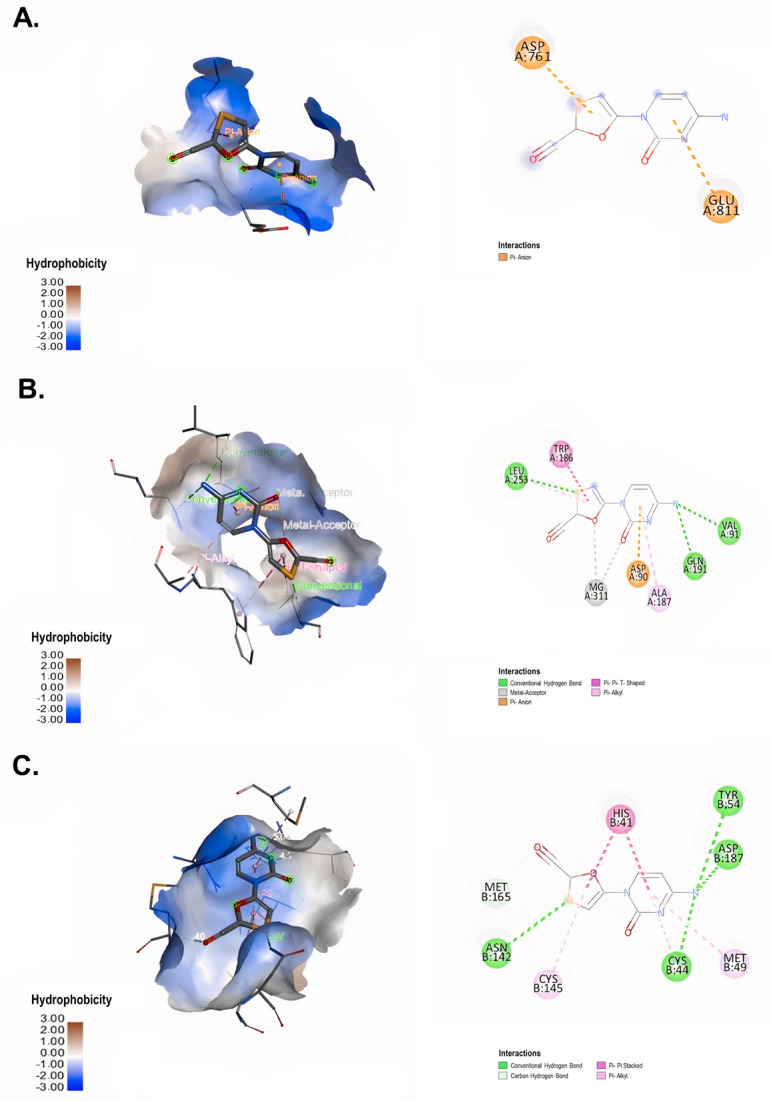
Interaction between 3TC and SARS-CoV-2 non-structural proteins according to molecular docking. 3D (left) and 2D (right) representative images of the main interaction of 3TC with (A) RdRp (PDB ID: 6M71), (B) ExoN-NSP10 (PDB ID: 7MC6) and (C) 3CLpro (PDB ID: 6M2N) through molecular docking. The types of interactions formed in the complexes are described in each image. The images were generated by using BIOVIA Discovery Studio Visualizer 16.1.

Four NRTIs (3TC, FTC, tenofovir and ABC) were evaluated in this study. The interaction between 3TC and RdRp yielded a binding energy of −4.9 kcal/mol (Table 2). This binding was generated by two π-anion bonds with the amino acids ASP 761 and GLU 811 of RdRp (Figure 3A). In contrast to RdRp, the interaction between 3TC and ExoN-NSP10 yielded a binding energy of −5.4 kcal/mol (Table 2). Additionally, 3TC formed three conventional hydrogen bonds with the amino acids LEU 253, VAL 91 and GLN 191 (distances of 3.68 Å, 3.29 and 3.29 Å, respectively), two hydrophobic interactions of π-Alkyl with LEU 253 and ALA 187, one hydrophobic interaction of π-π stacked with TRP 186 and electrostatic interaction with the amino acid ASP 90 of ExoN-NSP10 (Figure 3B). On the other hand, a binding energy of −5.8 kcal/mol was obtained from the SARS-CoV-2 3CLpro-3TC complex (Table 2). The amino acids MET 165, ASN 142, CYS 44, TYR 54 and ASP187 of 3CLpro formed conventional hydrogen bonds with 3TC (distances of 3.40 Å, 3.60 Å, 3.72 Å, 3.36 Å and 3.11 Å, respectively). Three hydrophobic interactions, such as π-π stacked with HIS 41 and π-alkyl bonds with CYS 145 and MET 49 of 3CLpro, were also found in this complex (Figure 3C).

**Figure 4.**
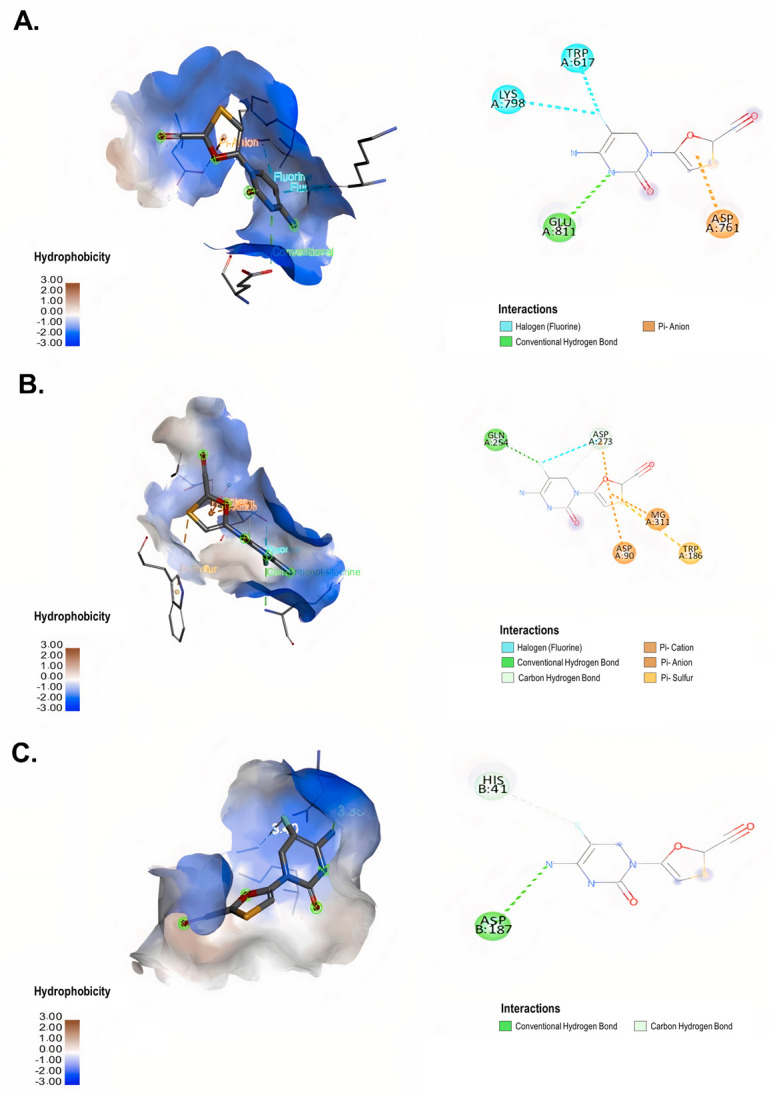
Interaction between FTC and SARS-CoV-2 non-structural proteins according to molecular docking. 3D (left) and 2D (right) representative images of the interaction of FTC with (A) RdRp (PDB ID: 6M71), (B) ExoN-NSP10 (PDB ID: 7MC6) and (C) 3CLpro (PDB ID: 6M2N) through molecular docking. The types of interactions formed in the complexes are described in each image. The images were generated by using BIOVIA Discovery Studio Visualizer 16.1.

In addition, FTC interacted with RdRp with a binding energy of −5.1 kcal/mol (Table 2). This compound formed a conventional hydrogen bond with GLU 811, hydrophobic interactions such as π-anion bonding with the amino acid ASP 761 and halogen interactions with LYS 798 and TRP 617 of RdRp (Figure 4A). The complex between FTC and ExoN-NSP10 showed a binding energy of −5.6 kcal/mol (Table 2). This interaction was established for conventional hydrogen bonds with the amino acids GLN 254 and ASP 273 (distances of 3.24 Å and 3.46 Å, respectively), two electrostatic interactions of π-anion with ASP 90 and ASP 273, a halogen interaction with ASP 273 and one π-sulfur interaction with TRP 186 of ExoN-NSP10 (Figure 4B). In contrast, a binding energy of −5.8 kcal/mol was obtained from the interaction between FTC and 3CLpro (Table 2). The amino acids ASP 187 and HIS 41 of 3CLpro formed conventional hydrogen bonds with FTC (distances of 3.35 Å and 3.40 Å, respectively) (Figure 4C).

Tenofovir and RdRp exhibited a binding energy of −4.9 kcal/mol (Table 2). In this complex, the amino acids ASP 761 and TRP 617 formed conventional hydrogen bonds with tenofovir. Further, electrostatic forces with ASP 618 and GLU 811 also participated in the stability of the RdRp-tenofovir complex (Supplementary Figure 1A). On the other hand, tenofovir and ExoN-NSP10 interacted with a binding energy of −5.7 kcal/mol (Table 2). This complex was established for four conventional hydrogen bonds with HIS 268, ASP 273, ASN 252 and GLN 254 (distances of 3.54 Å, 3.60 Å, 4.08 Å and 4.18 Å, respectively), two π-alkyl interactions with amino acids TRP 186 and ALA 267, one alkyl interaction with LEU 253 and three electrostatic interactions with ASP 273, GLU 92 and ASP 90 of ExoN-NSP10 (Supplementary Figure 1B). Further, a binding energy of −6.0 kcal/mol was obtained from the interaction between tenofovir and 3CLpro of SARS-CoV-2 (Table 2). The amino acids SER 144, CYS 145, TYR 54, CYS 44 and ASN 142 of 3CLpro formed five conventional hydrogen bonds with tenofovir (distances of 3.38 Å, 3.74 Å, 3.44 Å, 3.39Å and 3.64 Å, respectively). Electrostatic forces with GLU 166 also participated in the interaction of tenofovir with 3CLpro. Moreover, hydrophobic interactions such as π-stacked with HIS 41, π-alkyl with MET 49 and π-sulfur bonds with MET 165 of 3CLpro were observed in this complex (Supplementary Figure 1C).

Favorable binding energy was obtained between ABC and SARS-CoV-2 RdRp (−6.1 kcal/mol) in Table 2. In this complex, there were hydrophobic interactions such as π-alkyl bonds with the amino acids HIS 810, TRP 800 and LYS 798, as well as two π-anion bonds with the amino acids GLU 811 and ASP 761 of RdRp (Supplementary Figure 2A). In contrast, the interaction between ABC and ExoN-NSP10 yielded a binding energy of −6.8 kcal/mol (Table 2). In this complex, there were two conventional hydrogen bonds with the amino acids ASN 252 and HIS 268 (distances of 3.38 Å and 3.44 Å, respectively), two π-alkyl interactions with amino acids LEU 253 and ALA 267, one alkyl interaction with ALA 187, one π-sigma interaction with LEU 253 and electrostatic interactions of the π-anion type with ASP 90, GLU 92 and ASP 273 of ExoN-NSP10 (Supplementary Figure 2B). In addition, ABC obtained a binding energy of −6.8 kcal/mol with SARS-CoV-2 3CLpro (Table 2). This compound interacted with the amino acids HIS 164 and ASN 142 through conventional hydrogen bonds (distances of 3.38 Å and 4.03 Å, respectively). Moreover, hydrophobic interactions of the type π-sulfur with CYS 145 and MET 165, π-π stacked with HIS 41 and alkyl bonds with CYS 44 and MET 49 of 3CLpro were found in the 3CLpro-ABC complex (Supplementary Figure 2C).

Concerning non-NRTI (efavirenz), favorable binding energies were obtained with RdRp (−5.8 kcal/mol), ExoN-NSP10 (−7.1 kcal/mol) and 3CLpro (−6.2 kcal/mol) of SARS-CoV-2 (Table 2). Hydrogen bonds and hydrophobic interactions helped to stabilize these complexes. Efavirenz interacted with the amino acid ASP 618 of RdRp through a conventional hydrogen bond. Further, one alkyl bond with LYS 798 and halogen interactions with ASP 761, TRP 617 and GLU 811 of RdRp were observed (Supplementary Figure 3A). In contrast, efavirenz and ExoN-NSP10 formed two conventional hydrogen bonds with TRP 186 (distances of 2.85 Å and 3.42 Å, respectively), three alkyl interactions with amino acids ALA 267 and LEU 253 (two interactions with the last amino acid) and halogen interactions with ASP 273 and TRP 186 of ExoN-NSP10 (Supplementary Figure 3B). Conversely, efavirenz formed two conventional hydrogen bonds with ASN 142 (distances of 3.34 Å and 3.28 Å) and one with CYS 145 of 3CLpro (distance of 3.46 Å). This compound also formed three alkyl bonds with the amino acid MET 49 and one π-π stacked bond with HIS 41 of 3CLpro. Other interactions, such as π-sulfur and halogen bonds, were observed in the SARS-CoV-2 3CLpro-efavirenz complex (Supplementary Figure 3C).

**Figure 5.**
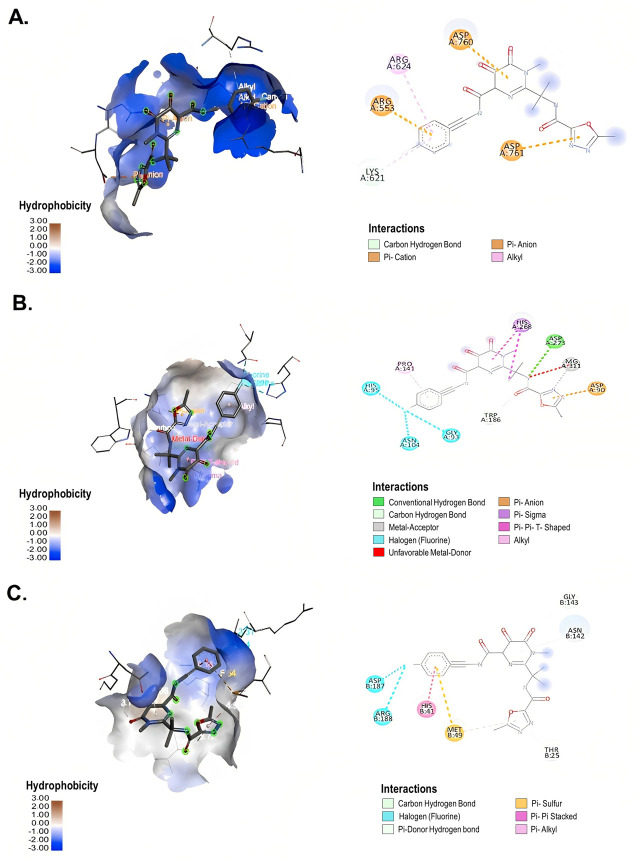
Interaction between RAL and SARS-CoV-2 non-structural proteins according to molecular docking. 3D (left) and 2D (right) representative images of the interaction of RAL with (A) RdRp (PDB ID: 6M71), (B) ExoN-NSP10 (PDB ID: 7MC6) and (C) 3CLpro (PDB ID: 6M2N) through molecular docking. The types of interactions formed in the complexes are described in each image. The images were generated by using BIOVIA Discovery Studio Visualizer 16.1.

The docking results showed that RAL, an integrase inhibitor, exhibited higher binding energies against RdRp, ExoN-NSP10 and 3CLpro of SARS-CoV-2 (−7.1 kcal/mol, −7.7 kcal/mol and −7.0 kcal/mol, respectively) than the other antiretrovirals evaluated (Table 2). This compound interacted with the amino acid LYS 621 of RdRp through one conventional hydrogen bond (distance of 3.34 Å). Further, hydrophobic interactions such as π-alkyl bonds with ARG 624 and LYS 621, π-cation bonds with ARG 553 and π-anion bonds with ASP 760 and ASP 761 of SARS-CoV-2 RdRp were observed (Figure 5A). In contrast to RdRp, RAL and ExoN-NSP10 formed two conventional hydrogen bonds with the amino acids ASP 273 and TRP 186 (distances of 3.07 Å and 3.51 Å, respectively), one alkyl interaction with PRO 141 and π-π T-shaped and π-sigma interactions with HIS 268. In addition, three halogen interactions with ASN 104, HIS 95 and GLY 93, as well as electrostatic π-anion interaction with ASP 90 of ExoN-NSP10, participated in this complex (Figure 5B). On the other hand, RAL formed three conventional hydrogen bonds with the amino acids ASN 142, GLY 143 and THR 25 of 3CLpro (distances of 4.06 Å, 3.78 Å and 3.72 Å, respectively). Furthermore, hydrophobic interactions such as π-π stacked bonds with HIS 41, π-alkyl and π-sulfur bonds with MET 49 and halogen bonds with ASP 187 and ARG 188 participated in the complex formed between RAL and SARS-CoV-2 3CLpro (Figure 5C).

This study included remdesivir, pibrentasvir and CQ as positive controls of interaction with RdRp, ExoN-NSP10 and 3CLpro of SARS-CoV-2, respectively. The interaction between remdesivir and RdRp yielded a binding energy of −7.1 kcal/mol (Table 2). Remdesivir formed 11 hydrogen bonds with the amino acids ASP 623 (distances of 3.88 Å and 3.10 Å), ARG 553 (3.27 Å), LYS 798 (3.27 Å), LYS 621 (3.38 Å and 2.92 Å), CYS 622 (3.33 Å and 3.40 Å), ASP761 (2.89 Å and 2.91 Å) and PRO 620 (3.39 Å). Hydrophobic interactions (π-anion, alkyl and π-alkyl bonds) and electrostatic interactions were also found in this complex.

Pibrentasvir and ExoN-NSP10 interacted with a binding energy of -6.6 kcal/mol (Table 2). Two conventional hydrogen bonds stabilized this complex with GLN 145 and GLN 254 (distances of 3.43 Å and 2.96 Å, respectively), hydrophobic interactions of π-alkyl with ALA 267, LEU 253, PHE 190 and PRO 141 and one π-π T-shaped interaction with HIS 95 were observed. In addition, halogen and metal-acceptor interactions were also found in this complex.

Finally, CQ and 3CLpro interacted with a binding energy of −6.3 kcal/mol (Table 2). This interaction was stabilized for 13 hydrogen bonds with HIS 164 (distances of 2.98 Å, 3.12 Å, 2.91 Å, 2.96 Å, 3.00 Å and 3.24 Å), HIS 163 (distances of 3.10 Å, 2.91 Å, 3.16 Å, 3.54 Å and 3.04 Å), MET 49 (2.81 Å) and CYS 44 (3.20 Å). Further, hydrophobic interactions such as π-alkyl, π-sigma and π-π T-shaped bonds were found in the 3CLpro-CQ complex.

According to the docking results, RAL had higher binding affinities with the non-structural proteins evaluated when compared to the other antiretrovirals tested. Interestingly, the interaction with ExoN-NSP10 showed a higher binding affinity for this compound. As shown in Table 2, all antiretrovirals, except RAL, showed higher binding affinities to the active sites of 3CLpro and ExoN-NSP10 when compared to the RdRp.

**Table 2.** Molecular docking of antiretrovirals and positive controls of interaction (remdesivir, pibrentasvir and CQ) with three non-structural SARS-CoV-2 proteins.

Ligand	Score with the pocket of viral proteins (kcal/mol)		
	RdRp (PDB ID: 6M71)	ExoN-NSP10 (PDB ID: 7MC6)	3CLpro (PDB ID: 6M2N)
Remdesivir	−7.1	−	−
Pibrentasvir	−	−6.6	−
Chloroquine	−	−	−6.3
Lamivudine	−4.9	−5.4	−5.8
Emtricitabine	−5.1	−5.6	−5.8
Tenofovir	−4.9	−5.7	−6.0
Abacavir	−6.1	−6.8	−6.8
Efavirenz	−5.8	−7.1	−6.2
Raltegravir	−7.1	−7.7	−7.0

## 4. Discussion

The rapid spread and high infectivity of SARS-CoV-2 and its potentially lethal and unpredictable course have prompted research on characterizing the virus and identifying efficient treatments and prophylactic alternatives [55],[56]. In this study, the antiviral activity of antiretroviral drugs was evaluated against SARS-CoV-2 in vitro and in silico.

Nucleoside analogues are widely used for the treatment of viral infections such as hepatitis B virus [57], HIV [58], hepatitis C virus [59], herpes simplex virus [60], Ebola virus [30] and SARS-CoV [42]. Two nucleoside analogues evaluated in this study (3TC and FTC) inhibited infectious SARS-CoV-2 particles on Vero E6 cells (Figure 2). According to these results and the antiviral mechanism reported for these drugs [61], we evaluated their interaction with SARS-CoV-2 RdRp through molecular docking. Similar to the report by Copertino et al. [26], our results showed that both drugs bound with low affinity to the active site of this enzyme through conventional hydrogen bonds or hydrophobic interactions such as the π-anion bond and halogen interactions (Table 2). These findings are in accordance with the report by Chien et al. [27], who demonstrated through polymerase extension experiments that 3TC and FTC could be incorporated by SARS-CoV-2 RdRp, with varying efficiency [27].

Additionally, previous studies have indicated that some nucleoside analogues inhibit coronavirus ExoN activity [62],[63]. In this study, we identified favorable interactions of the ExoN-NSP10 complex of SARS-CoV-2 with 3TC and FTC by using bioinformatic methods (Table 2). These results suggest that both drugs, in addition to an inhibition of RdRp, could bind with low affinity to the ExoN-NSP10 complex. Previous reports have suggested that NRTIs could affect viral replication via direct inhibition of ExoN, incorporation into the elongation reaction faster than the ExoN cleavage reaction or staying at the nascent chain by not being recognized as ExoN [52],[63]. However, experimental assays are needed to confirm the activity of 3TC and FTC against this viral enzyme.

On the other hand, Tran et al. reported favorable affinity (between −5.8 and −8.8 kcal/mol) of NRTIs with SARS-CoV-2 3CLpro [64], one of the most attractive viral targets for an antiviral drug against this virus [65]. Therefore, we also evaluated the binding affinity of 3TC and FTC with the proteolytic site of 3CLpro, obtaining favorable binding energies, although low, for both compounds (Table 2). The complexes formed between 3CLpro with 3TC and FTC were stabilized by conventional hydrogen bonds and hydrophobic interactions such as π-π stacked and π-alkyl bonds. These results suggest that these compounds could interact with several viral targets.

Due to the absence of reference compounds to evaluate the interaction with SARS-CoV-2 proteins through molecular docking, we selected as positive controls those compounds that previously demonstrated a high binding affinity to RdRp ExoN-NSP10 and 3CLpro in the literature [44]–[48]. According to our in silico results, NRTIs and NNRTIs showed a lower binding affinity to the viral proteins than the positive interaction controls (Table 2). Only ABC and efavirenz showed the comparable or higher binding affinity to ExoN-NSP10 and/or 3CLpro than the positive controls (pibrentasvir and CQ, respectively) (Table 2).

Despite the above, in vitro findings indicated that ABC and efavirenz did not exhibit antiviral activity against SARS-CoV-2 under our tested conditions (Figure 2). The differences between the in silico and in vitro results could be due to molecular docking using approximations that do not accurately take into account some thermodynamic elements of the binding energy, such as entropy changes upon binding and the influence of the solvent [66]–[68], thus, a complete correlation with the experimental results was not expected.

These differences could also be explained by specific transport systems for the entry to the cells, intracellular metabolism or export mechanisms of each compound, which could affect the accessibility of tested drugs to viral targets within infected cells [69],[70]. Concerning mammalian cells, the entry of nucleoside analogues occurs through the nucleoside transport systems that facilitate diffusion or actively transport nucleosides across the membrane; therefore, the specificity of each compound uptake by transporter proteins determines its access to intracellular compartments and, consequently, affects its biological activity [71].

In addition, each type of cell expresses different activation mechanisms involved in nucleoside, nucleotide and pyrophosphate analogue metabolisms, which can induce differences in their antiviral activity [72]. A previous study found that remdesivir, an adenosine analogue, exhibited less antiviral activity against SARS-CoV-2 in Vero E6 as compared to other cell lines. This difference was associated with a lower intracellular concentration of the pharmacologically active metabolite in Vero E6 [69]. This finding reflects the need to evaluate the antiviral effect of antiretrovirals in other cell lines permissible to infection.

On the other hand, it has been previously reported that Vero cells express high levels of the efflux transporter P-glycoprotein encoded by MDR1 or ABCB1 [73], which efficiently exports nucleoside analogues and, thereby, can limit the therapeutic efficacy of these compounds [74].

Regarding NRTIs, different clinical studies are being developed to evaluate the efficacy of FTC in combination with tenofovir with the aim of preventing or treating COVID-19 disease [75]–[77]. A randomized pilot phase-two trial developed in France found that an FTC and TDF combination appeared to accelerate the natural clearance of nasopharyngeal SARS-CoV-2 viral burden among outpatients adults with recent non-severe COVID-19 [78]. Another study conducted in Colombia found less mortality in hospitalized patients with pulmonary compromise from COVID-19 after 28 days of treatment with FTC in combination with TDF (mortality of 13.8%), and both in combination with tenofovir disoproxil plus colchicine and rosuvastatin (mortality of 10.7%) [79]. Finally, Del Amo et al. found that HIV-positive patients with FTC and TDF treatment had a lower risk for COVID-19 and related hospitalization than those receiving other therapies (tenofovir alafenamide and FTC or ABC/3TC) [80]. These findings support future research on the effect of antiretroviral combinations for treating COVID-19 in individuals with and without HIV.

Additionally, we identified the in vitro antiviral effect of RAL against SARS-CoV-2. This drug was the first approved HIV-1 integrase inhibitor, and its antiviral activity against retrovirus human T-cell lymphotropic virus type 1 was also demonstrated. RAL acts by binding to the active site of the integrase, preventing viral DNA insertion into the host genome [81]. Although SARS-CoV-2 lacks an enzyme with this function, it has been previously reported that integrase inhibitors could interact with non-structural SARS-CoV-2 proteins (3CLpro [82], RdRp [83], NSP14 and NSP16 [20],[84]), suggesting that this type of compound could have antiviral mechanisms against SARS-CoV-2 that are different from that reported for HIV. Our in silico results showed that RAL had better binding affinities with the non-structural proteins evaluated (RdRp, ExoN-NSP10 and 3CLpro) than other antiretrovirals tested and positive controls of interaction; the interaction with ExoN-NSP10 was the one to exhibit a higher binding affinity (Table 2). These findings are related to the those reported by Baddock et al. [10]; they found that RAL inhibits the exonuclease activity of NSP14-NSP10 (half-maximal inhibitory concentration value of 24.4 ± 2.7 µM) through a gel-based nuclease assay [10]. However, additional studies are required to elucidate its antiviral mechanism and its effectiveness (as monotherapy or in combination with other drugs) in preventing or treating COVID-19.

Our study only evaluated in silico interactions of the compounds against the active sites of the viral proteins. Therefore, possible interactions of antiretrovirals with other protein pockets related to the allosteric impairment of protein function should be evaluated in further studies. In addition, the effects of these compounds on cellular targets, such as nucleoside transporters or cellular kinases that affect the accessibility, metabolism or intracellular accumulation of these drugs, should be determined. Concerning in vitro methodology, the evaluation of the antiviral activity against SARS-CoV-2 in human cell lines and new emergent variants is necessary.

## 5. Conclusion

In summary, 3TC, FTC and RAL showed antiviral effects against the D614G strain of SARS-CoV-2 in vitro. RAL was the compound with the greatest in vitro antiviral potential at low concentrations, and it showed the highest binding affinities with crucial SARS-CoV-2 proteins during the viral replication cycle. Further studies are required to examine the therapeutic utility of this compound in COVID-19 patients.
