# Supplementary material for: In vitro and in silico evaluation of antiretrovirals against SARS-CoV-2: A drug repurposing approach
Source: AIMS Microbiol. 2023 Jan 16;9(1):20–40. doi: 10.3934/microbiol.2023002 (PMC9988408; doi:10.3934/microbiol.2023002)
Supplement: Supplementary file 1 [file microbiol-09-01-002-s001.pdf]

AIMS Microbiology

AIMS Microbiology, 9(1): 20–40.
DOI: 10.3934/microbiol.2023002
Received: 05 September 2022
Revised: 26 November 2022
Accepted: 13 December 2022
Published: 12 January 2023

http://www.aimspress.com/journal/microbiology

*Research article*

# In vitro and in silico evaluation of antiretrovirals against SARS-CoV-2: A drug repurposing approach

**Maria I. Zapata-Cardona[1], Lizdany Florez-Alvarez[1,2], Ariadna L. Guerra-Sandoval[3], Mateo Chvatal-Medina[1], Carlos M. Guerra-Almonacid[3], Jaime Hincapie-Garcia[4], Juan C. Hernandez[5], Maria T. Rugeles[1] and Wildeman Zapata-Builes[1,5,*]**

[1] Grupo Inmunovirologia, Facultad de Medicina, Universidad de Antioquia UdeA, Medellin, Colombia
[2] Institute of Biomedical Sciences, University of Sao Paulo, Sao Paulo, Brazil
[3] Grupo de investigacion GIRYSOUT, Universidad del Tolima, Ibague, Colombia
[4] Grupo de investigacion, Promocion y prevencion farmaceutica, Facultad de ciencias farmaceuticas yalimentarias, Universidad de Antioquia UdeA, Medellin, Colombia
[5] Grupo Infettare, Facultad de Medicina, Universidad Cooperativa de Colombia, Medellin, Colombia

* **Corresponding:** Email: wildeman.zapatab@campusucc.edu.co.

## Supplementary

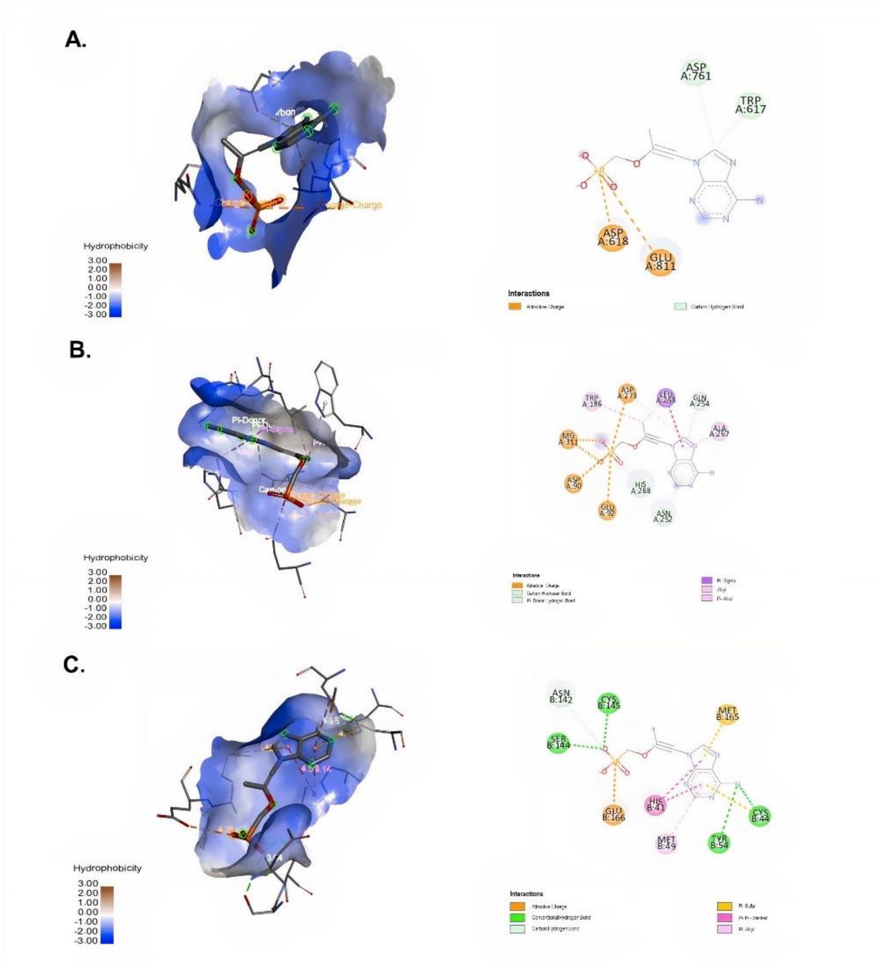


**Figure S1.** Interaction between tenofovir and SARS-CoV-2 non-structural proteins, using molecular docking. 3D (Figure left) and 2D (Figure right) representative images of the interaction of tenofovir with (A) RdRp (PDB ID:6M71), (B) ExoN-NSP10 (PDB ID:7MC6) and (C) 3CLpro (PDB ID:6M2N) by molecular docking. The types of interactions formed in the complexes are described in each Figure. The images were generated by using BIOVIA Discovery Studio Visualizer 16.1.

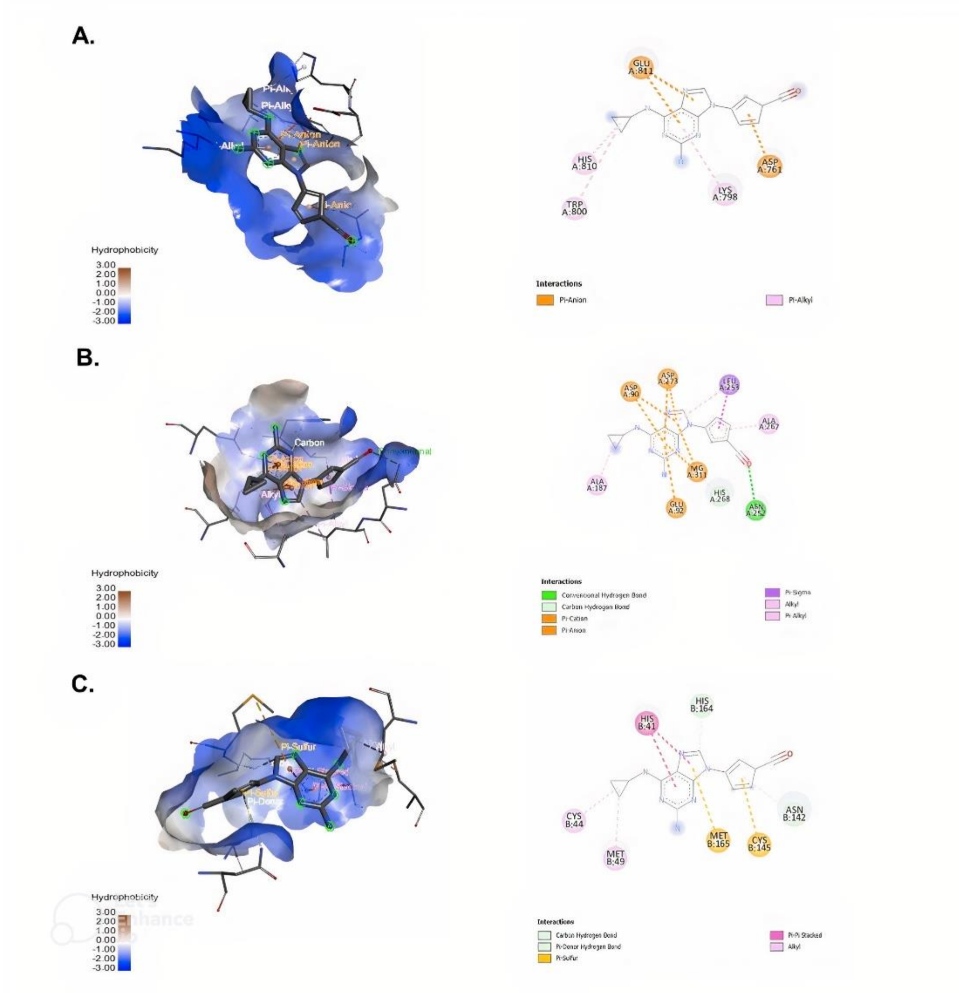


**Figure S2.** Interaction between abacavir and SARS-CoV-2 non-structural proteins, using molecular docking. 3D (Figure left) and 2D (Figure right) representative images of the interaction of abacavir with (A) RdRp (PDB ID:6M71), (B) ExoN-NSP10 (PDB ID:7MC6) and (C) 3CLpro (PDB ID:6M2N) by molecular docking. The types of interactions formed in the complexes are described in each Figure. The images were generated by using BIOVIA Discovery Studio Visualizer 16.1.

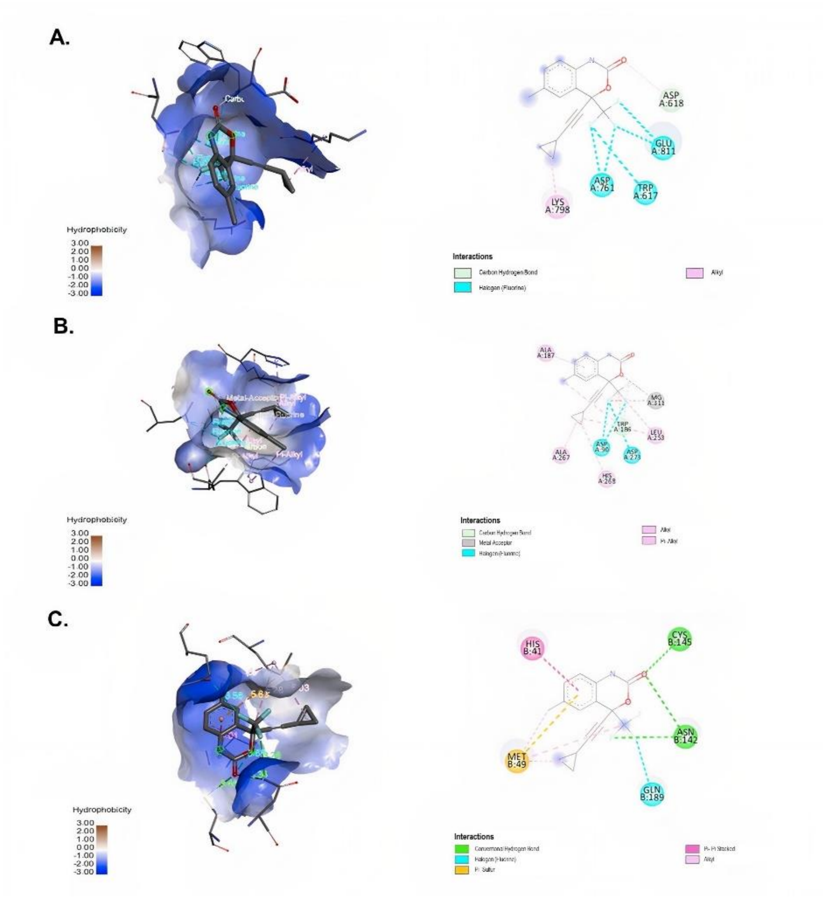


**Figure S3.** Interaction between efavirenz and SARS-CoV-2 non-structural proteins, using molecular docking. 3D (Figure left) and 2D (Figure right) representative images of the interaction of efavirenz with (A) RdRp (PDB ID:6M71), (B) ExoN-NSP10 (PDB ID:7MC6) and (C) 3CLpro (PDB ID:6M2N) by molecular docking. The types of interactions formed in the complexes are described in each Figure. The images were generated by using BIOVIA Discovery Studio Visualizer 16.1.
